# Genome-scale models of bacterial metabolism: reconstruction and applications

**DOI:** 10.1111/j.1574-6976.2008.00146.x

**Published:** 2008-12

**Authors:** Maxime Durot, Pierre-Yves Bourguignon, Vincent Schachter

**Affiliations:** Genoscope (CEA) and UMR 8030 CNRS-Genoscope-Université d'Evry, EvryFrance

**Keywords:** metabolic network, systems biology, computational methods, genome-scale metabolic models, metabolic engineering, omics data integration

## Abstract

Genome-scale metabolic models bridge the gap between genome-derived biochemical information and metabolic phenotypes in a principled manner, providing a solid interpretative framework for experimental data related to metabolic states, and enabling simple *in silico* experiments with whole-cell metabolism. Models have been reconstructed for almost 20 bacterial species, so far mainly through expert curation efforts integrating information from the literature with genome annotation. A wide variety of computational methods exploiting metabolic models have been developed and applied to bacteria, yielding valuable insights into bacterial metabolism and evolution, and providing a sound basis for computer-assisted design in metabolic engineering. Recent advances in computational systems biology and high-throughput experimental technologies pave the way for the systematic reconstruction of metabolic models from genomes of new species, and a corresponding expansion of the scope of their applications. In this review, we provide an introduction to the key ideas of metabolic modeling, survey the methods, and resources that enable model reconstruction and refinement, and chart applications to the investigation of global properties of metabolic systems, the interpretation of experimental results, and the re-engineering of their biochemical capabilities.

## Introduction

The flow of genome sequencing, metagenome sequencing and other high-throughput experimental efforts aimed at exploring the space of microbial biochemical capabilities has been steadily growing in recent years. At the time of writing, more than 1800 bacterial genome-sequencing projects have been initiated and nearly 650 have been completed (http://www.genomesonline.org, http://www.ebi.ac.uk/integr8). Combined with increasingly efficient annotation methods, these set the stage for the systematic identification of most enzymes encoded in the genomes of the corresponding bacterial species. A variety of so-called ‘-omics’ technologies now routinely provide large-scale functional clues on molecular interactions and cellular states, offering snapshots of the dynamic operation of metabolism under specified conditions, and adding to the store of accumulated knowledge on microbial biochemistry and physiology.

Simultaneously, the expected wealth of new biochemical activities, the progress of metabolic engineering techniques aimed at harnessing these activities, and the perspective of applications to white and green biotechnology have triggered a strong renewed interest in the exploration of bacterial metabolism. In addition to charting the range of naturally evolved chemical transformations, relevant research questions include the following: How does the global metabolism of a bacterium react to changes in its environment? What kind of joint metabolic operation of distinct species can help sustain a bacterial community? How can genomic and biochemical information be best exploited to gain insights into the relationship between an organism's genotype and its phenotype? For instance, can we predict changes in metabolism-related phenotypic traits caused by simple or complex genotype modifications? How did metabolic processes evolve? How can metabolic networks be efficiently reprogrammed for a variety of utilitarian purposes?

Investigations of a bacterium's metabolism are typically fed by knowledge (ultimately from observations) at two different scales of description of the chemistry at work within cells. The larger scale focuses on the physiology of the whole bacterial cell. For instance, which media is it able to grow on? What are the relative quantities of chemical nutrients it requires for growth? How efficient is the cell at converting chemicals from the environment into its own components? Such metabolic capabilities result from the coordinated action of the enzymes expressed in the respective species, the knowledge of which belongs to the finer, molecular scale. Each of the corresponding biochemical conversions can be identified either directly by performing enzymatic assays, or indirectly, from the genome sequence, through a homology relationship with proteins whose function has been previously elucidated. Together, the reactions that have been demonstrated to potentially occur in the cell form the *metabolic network* of the organism. Metabolic networks can thus be viewed as lists of those molecular mechanisms (reactions) and associated molecular components (enzymes, substrates, and products) that are most directly related to the metabolic capabilities mentioned above.

For a given bacterial species, confronting knowledge from these two scales, molecular vs. cellular, can reveal inconsistencies. For instance, it may happen that no sequence of identified reactions is capable of producing one of the essential cell components from the set of compounds available in a defined growth medium, even though the species is known to grow on that medium. Furthermore, when the two scales are consistent, their relationship can be investigated further in order to enumerate the possible implementations of the physiology that the metabolic network can achieve. Biochemists have traditionally performed such investigations by modularizing the set of reactions into *metabolic pathways*, typically grouping together reactions that allow the conversion of one or more ‘input’ metabolites into ‘output’ metabolites. Pathways boundaries are somewhat arbitrary, even though inputs and outputs tend to be metabolites involved in several reactions. Pathway-based analyses are thus focused on the possible fates of a restricted number of compounds, and are amenable to manual expertise thanks to the simplification brought by the modularized view (Huang *et al.*, 1999; Teusink *et al.*, 2005; Risso *et al.*, 2008).

Yet, metabolic pathways typically involve a large number of ‘side metabolites’ such as cofactors and byproducts of chemical reactions, and metabolism is as much about converting nutrient into cell components as it is about regenerating cofactors and recycling (or secreting) ultimately unused byproducts. The latter transformations typically involve several pathways, and are dependent on the stoichiometry and rates of the reactions. Manual approaches are insufficient to assess their feasibility by a given network for at least two reasons: metabolic networks are too large, and the question requires a quantitative analysis.

Bridging that gap between knowledge of the metabolic network structure and observed metabolic phenotypes is precisely where metabolic models come into play. Generally speaking, a model of a natural system is one of many possible mathematical representation of that system, explicitly describing some of its features and supporting predictions on some other features, the latter being typically time- or environment dependent. In this particular case, knowledge of the metabolic network alone is not quite sufficient to predict the metabolic capabilities of a cell. Also needed are a structured (mathematical) representation of that network, together with a set of rules and possibly quantitative parameters enabling simulations or predictions on the joint operation of all network reactions in a given environment, and in particular predictions on the values of metabolite fluxes and/or concentrations (Papin *et al.*, 2003). The above, in short, constitutes a metabolic model.

Constraint-based genome-scale models of metabolism (Palsson, 2006) are a category of models precisely aimed at assessing the physiological states achievable by a given metabolic network, and at uncovering their biochemical implementation in terms of metabolic fluxes. They offer an idealized view of the cell as a set of ‘pipes,’ with metabolites flowing through each pipe, and biochemical conversions taking place at junctions between pipes. Some metabolites can also be exchanged with the environment, flowing in or out of the system through dedicated pipes that can be opened or shut, and may have upper bounds on their throughput. The cell is required to achieve balanced production and consumption of all the intermediate substrates and products involved in its metabolism: what flows in a junction must flow out.

Constraint-based models can help investigate in a systematic manner most of the research questions listed at the start of this introduction, because they provide a way to explore the consequences on the operation of the entire metabolic network of the piecemeal information available on each of its parts. They are especially well suited to ‘what if’ experiments involving genetic or environmental perturbations, such as: how would the cell behave in an environment with a different chemistry than the ones that have been experimented on? How would one or more deletions affect its metabolic capabilities? Which deletions would maximize the production of both metabolite *x* and biomass?

Before a model for a given species can be used to gain new insights into its metabolic capabilities or evolutionary history, it must first be built from the scattered genomic, biochemical, and physiological information available on that species up to a point where known physiology can be predicted from biochemistry without major mistakes. This process is sometimes known as ‘model reconstruction’; its endpoint is a functional genome-scale model, i.e. a structured representation of the current state of knowledge on the metabolism of the respective species (Reed *et al.*, 2006a). The model provides a framework to interpret new experimental data gathered at the cellular or molecular scale. That data may be incompatible with the current model, in which case either or both should be questioned, leading to possible revisions or improvements. If, on the other hand, data and model are compatible, the new evidence may still narrow down the set of possible metabolic behaviors of the cell, thus enriching the model (Covert *et al.*, 2004).

This review article covers both the reconstruction of genome-scale metabolic models and their applications to basic and applied research in microbiology. Following a primer on constraint-based models, we will review the state of the art in model reconstruction. Next, we will survey the main applications of metabolic models, from phenotype predictions to data interpretation or metabolic engineering. Practical aspects of direct relevance to the working microbiologist will be covered by a sketch of the main dedicated database and software resources. We will conclude the review with a discussion on future directions in the field.

## Foundations of genome-scale metabolic modeling

The metabolic state of a cell and its variation over time can be described by metabolite concentrations and reaction rates, which can be viewed as the ‘endpoints’ of metabolic operation. These quantities are related by the law of conservation of matter, which states that the net production rate of a metabolite equals the sum of the rates of the reactions consuming or producing it, weighted by the associated relative stoichiometric coefficients. Conversely, enzyme kinetics express reaction rates as complex functions of metabolite concentrations and enzymatic activities, which vary over time as a result of transcriptional and metabolic regulation (Smallbone *et al.*, 2007). Deriving meaningful predictions from these two types of equations for large metabolic systems is a very challenging proposition, not only because of the mathematics, but also because many of the parameters are not known, difficult to measure, and possibly context dependent. In practice, these pitfalls restrict the use of kinetic modeling to metabolic systems much smaller than ‘whole-cell’ metabolic networks, which typically include hundreds of reactions for a bacterium.

Constraint-based models bypass these difficulties by focusing on the average reaction rates achievable by cells grown in steady or slowly varying environmental conditions. Rates are typically averaged over minutes, fitting with the typical time scale of uptake or secretion rates measurements. Such averages are not affected by transient states because the characteristic relaxation time of metabolic systems – i.e. the time it takes for chemical reactions within the cell to reach a steady state – is much shorter than a minute. Moreover, because environmental changes and variations of enzyme concentrations occur on longer time scales, one need not take into account regulatory changes to assess average reaction rates over minutes. Turnover rates of most intracellular metabolites are high in bacterial cells (Stephanopoulos *et al.*, 1998). At the time scale considered here, their concentrations have therefore generally reached steady levels, and remain constant as long as environmental conditions do not change. As a consequence, the law of conservation of matter constrains the production and consumption rates of these metabolites to be balanced. These assumptions are usually summarized under the expression *steady-state hypothesis* and the corresponding constraint on reaction rates as a *mass balance* (or stoichiometric) *constraint* (Stephanopoulos *et al.*, 1998). Obviously, this reasoning applies only to metabolites that are neither taken in from an external pool (e.g. nutrients) nor excreted from the cell or accumulated in large quantities (e.g. cell components such as nucleic acids, amino acids, or some lipids). For each metabolite that can be ‘balanced,’ the mass balance constraint can be expressed mathematically by a linear equation relating reaction rates of the form ∑*s*_*j*_*ν*_*j*_=0, where *s*_*j*_ is the stoichiometric coefficient of the metabolite in reaction *j*, and *ν*_*j*_ the rate of reaction *j*.

In addition to mass balance constraints, reactions that are known to be thermodynamically irreversible *in vivo* are constrained to have a non-negative reaction rate. Similarly, upper bounds on the reaction rates can be known from measurements or theory and included in the model as additional constraints on the reaction fluxes (Reed & Palsson, 2003).

**Fig. 1:**
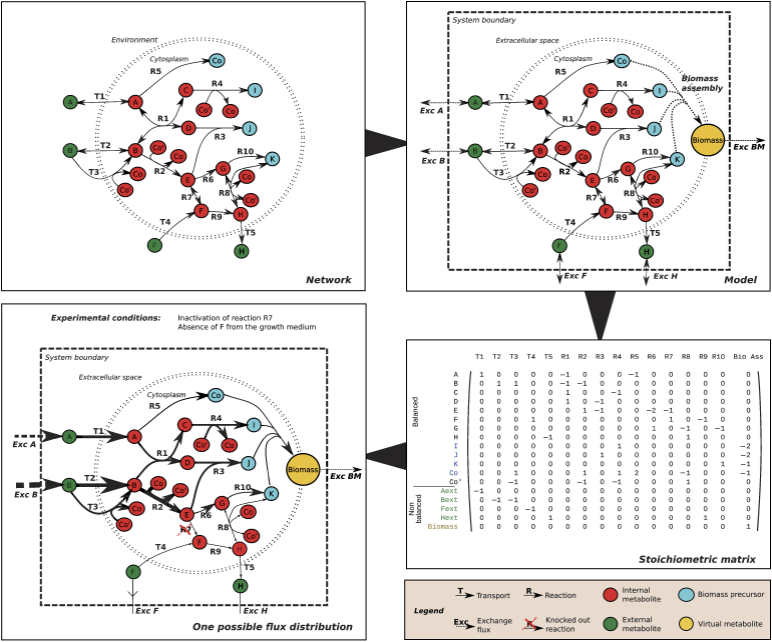
Genome-scale modeling of metabolism. A metabolic network (top left) is transformed into a model by defining the boundaries of the system, a biomass assembly reaction, and exchange fluxes with the environment (top right). Using the corresponding stoichiometric matrix (bottom right), the achievable flux distributions compatible with enforced constraints can be found (a particular one is depicted in the bottom left figure).

Mass balance, irreversibility and upper-bound constraints result from the application of simple laws of physics to individual reactions or metabolites from the network. These constraints propagate from reaction to reaction throughout the metabolic network; the constraint-based modeling framework is designed to automatically compute the resulting balance. To that end, it makes use of a succinct mathematical representation of all reaction stoichiometries: the *stoichiometric matrix* (see Fig. 1). In this matrix, columns represent reactions and rows metabolites. The stoichiometric coefficient of a metabolite within a reaction is included at the intersection of the corresponding row and column (see Fig. 1). Reaction rates are represented in constraint-based models by single numbers, the *reaction fluxes*, which are normalized by the weight of the cells harboring the reactions to account for the size of the colony (a reaction flux is typically expressed with the Unit mmol h^−1^ g^−1^ dry wt). Because the goal is to describe the joint operation of many metabolic reactions, it is convenient to define a *flux distribution* as a collection of reaction fluxes covering the entire system. Under the steady-state approximation, the concentrations of balanced metabolites being constant, a flux distribution carries sufficient information to completely describe a state of the system. Using the stoichiometric matrix, a simple matrix equation – summarizing all mass balance equations shown above – can then be used to enforce the mass balance constraints on all reactions fluxes: *S.ν*=0, where *S* is the stoichiometric matrix and *ν* the flux distribution represented as a vector.

A precise definition of the boundary of the system to be modeled is also needed to formulate an explicit mathematical representation. The system typically includes the whole cell and its vicinity, in order to encompass all the exchanges of matter between the cell and its environment. Transport reactions that allow for exchange of specific metabolites with the extracellular space through the membrane are also included in the model. Environmental conditions are then modeled by acting on the balance of the external metabolites: metabolites that are available from the environment can be taken up by transporters while the others can only be excreted.

A flux distribution that is compatible with all the constraints in a given environment is considered achievable (or feasible) by the cell, whereas a distribution that violates at least one of these constraints is not. The simplicity of the system of linear equations that represent constraints is one of the main strengths of the framework, because it permits fast assessments of the feasibility of a flux distribution using a computer and standard algorithms.

The simplicity of constraint-based models comes at the expense of a number of limitations in their predictive capabilities. Such models focus solely on reaction fluxes, and completely ignore the influence of metabolites and enzymes. In reality, however, enzyme kinetics, and transcriptional or metabolic regulation may significantly influence reaction fluxes. Regulation can for instance limit the use of a pathway by downregulating some of its enzymes when particular environmental conditions are met. These mechanisms, if they could somehow be taken into account, would eliminate flux distributions otherwise allowed by constraint-based models. In other words, models may allow ‘false-positive’ metabolic states, which respect the enforced metabolic constraints but are inconsistent with other biological processes. Several attempts have been made to extend the constraint-based modeling framework, in order to account for regulatory interactions (Covert *et al.*, 2001), signaling processes (Lee *et al.*, 2008b), the first and second laws of thermodynamics (Beard *et al.*, 2002, 2004), or metabolite concentrations (Kümmel *et al.*, 2006b; Henry *et al.*, 2007). Nevertheless, these extensions require the inclusion of additional experimental data and may result in more complex mathematical formulation hindering their practical use.

Some predictions of constraint-based models may be wrong in cases where modeling assumptions do not hold. For instance, some metabolites do accumulate in the cell, and the mass balance assumption clearly does not hold for these. In general, the concentration of specific metabolites may be high enough relatively to the fluxes they are involved in for the mass balance approximation to become clearly false.

In practice, many of the analytical methods that have been developed for constraint-based models focus on defining and characterizing sets of feasible flux distributions. Others focus on a single distribution. The diversity of flux distributions compatible with constraints in a given environment can be viewed as reflecting the diversity of the metabolic states the cell may find itself in. Nevertheless, the space of feasible flux distributions features biologically informative properties whose determination requires adequate techniques; these will be introduced in the next sections of this review.

## Building the models

The level of detail necessary to build a constraint-based model of a bacterium's metabolism is relatively low; the only information required is the precise reaction stoichiometries and directions, in order to account for mass balance and irreversibility constraints. To reflect the global biochemical capabilities of the organism, the model also needs to encompass the complete set of metabolic activities that can occur within it – or a reasonable approximation thereof. This comprehensiveness requirement and the high number of metabolic reactions make the actual construction of such models a challenging task in itself. In this section, we will review the main methods and resources helping in this task. We will first show how information from genome annotation can be used to infer biochemical reactions at large scale, a task commonly called *metabolic network reconstruction*. We will then review the techniques commonly used to assess the consistency of reconstructed models, and show how missing biochemical activities can be identified to complete the model.

### Initial reconstruction of metabolic models

The most reliable evidence from which the presence of a metabolic reaction in a species can be inferred is experimental proof of the respective biochemical activity. Such biochemical results have been accumulated for several decades, mostly from dedicated experiments targeting well-defined activities. As a consequence, the corresponding reactions have often been precisely and reliably characterized. Exploiting these results to reconstruct the whole metabolism of an organism is a labor-intensive task, however, as it requires processing a high volume of literature. Most existing metabolic models have been reconstructed in this manner and for extensively studied organisms. For instance, the most complete bacterial model available to date – namely iAF1260, the latest model of *Escherichia coli* metabolism – includes references to more than 320 articles (Feist *et al.*, 2007). Two types of databases centralize biochemical knowledge: enzyme-centric ones, which collect functional information acquired on enzymes, for example BRENDA (Barthelmes *et al.*, 2007) or SwissProt (Boutet *et al.*, 2007); and pathway databases, aimed at describing the biochemistry of metabolic processes, for example EcoCyc for *E. coli* metabolism (Karp *et al.*, 2007) or UM-BDD for microbial biodegradation pathways (Ellis *et al.*, 2006) (see Table 1).

**Table 1:** Data sources for metabolic model reconstruction and refinement

**DNA sequence and genome annotation databases**		
DDBJ	http://www.ddbj.nig.ac.jp/	General nucleotide sequence database
EMBL	http://www.ebi.ac.uk/embl/	General nucleotide sequence database
GenBank	http://www.ncbi.nlm.nih.gov/Genbank/	General nucleotide sequence database
Integr8	http://www.ebi.ac.uk/integr8/	Integrated information on complete genomes
CMR	http://cmr.jcvi.org/	Integrated information on complete prokaryotic genomes
IMG	http://img.jgi.doe.gov/	Integrated system for analysis and annotation of microbial genomes
SEED	http://seed-viewer.theseed.org/	Integrated system for analysis and annotation of genomes using functional subsystems
**Protein and enzyme databases**		
BRENDA	http://www.brenda-enzymes.info/	Comprehensive enzyme information system gathering data collected from the literature by curators
ENZYME	http://www.expasy.ch/enzyme/	Enzyme nomenclature database providing extensive information on all enzymes with an associated EC number
UniProt	http://www.ebi.ac.uk/uniprot/	Universal Protein Resource gathering protein sequences and annotations from SwissProt (manually reviewed), trEMBL (computer annotated), and PIR
TransportDB	http://www.membranetransport.org/	Predictions of membrane transport proteins for fully sequenced genomes
PSORTdb	http://db.psort.org/	Repository of experimentally determined and predicted protein localizations
Prolinks	http://prolinks.mbi.ucla.edu/	Database of predicted functional links between proteins
STRING	http://string.embl.de/	Database of known and predicted protein–protein interactions
**Metabolic databases**		
CheBI	http://www.ebi.ac.uk/chebi/	Database on small molecules of biological interest
Pubchem	http://pubchem.ncbi.nlm.nih.gov/	Database on small molecules
LipidMaps	http://www.lipidmaps.org/	Database on lipid metabolites
Reactome	http://www.reactome.org/	Curated database of biological pathways
KEGG	http://www.genome.jp/kegg/	Suite of databases comprising information on compounds, reactions, pathways, genes/proteins
BioCyc	http://www.biocyc.org/	Collection of organism-specific pathway/genome databases, including a curated multiorganism pathway database: MetaCyc
UniPathway	http://www.grenoble.prabi.fr/obiwarehouse/unipathway/	Curated resource of metabolic pathways linked to UniProt enzyme database
UM-BBD	http://umbbd.msi.umn.edu/	Database on microbial biocatalytic reactions and biodegradation pathways
**Experimental data repositories**		
IntAct	http://www.ebi.ac.uk/intact/	Repository of reported protein interactions
DIP	http://dip.doe-mbi.ucla.edu/	Database of experimentally determined interactions between proteins
Array Express	http://www.ebi.ac.uk/aerep/	Public repository of microarray data
GEO	http://www.ncbi.nlm.nih.gov/geo/	Public repository of microarray data
ASAP	http://asap.ahabs.wisc.edu/	Repository of results of functional genomics experiments for selected bacterial species
*E. coli* multi-omics DB	http://ecoli.iab.keio.ac.jp/	Comprehensive dataset of transcriptomic, proteomic, metabolomic, and fluxomic experiments for *E. coli* K12
Systomonas	http://www.systomonas.de/	Repository of ‘omics’ datasets and molecular networks for pseudomonads species
PubMed	http://www.pubmed.org/	Database on biomedical literature
**Metabolic model repositories**		
BiGG	http://bigg.ucsd.edu/	Repository of reconstructed genome-scale metabolic models
BioModels	http://www.ebi.ac.uk/biomodels/	Database of mathematical models of biological systems

These biochemical clues are typically incomplete relatively to the set of all possible activities, especially for less studied organisms. In addition, while technologies aiming at high-throughput characterization of biochemical activities are improving, they are not yet mature enough to provide reasonably good coverage. Genes corresponding to enzymes that have been experimentally characterized have nevertheless been identified. Their homologues in the genome of such species can be identified using comparative genomics methods, thereby indicating the presence of the associated biochemical activities.

The traditional path to inferring metabolic reactions from the genome of an organism is gene-centric, at least in its first steps. Nearly all available genome sequences are now systematically processed through automated annotation pipelines, which identify coding sequences and infer functional annotations. Covering all relevant methods would be beyond the scope of this article, but thorough reviews can be found elsewhere (Médigue & Moszer, 2007). Basically, coding sequences are first identified using highly efficient gene-finding algorithms [such as genemark (Besemer *et al.*, 2001), glimmer (Delcher *et al.*, 1999), or amigene (Bocs *et al.*, 2003)], which discard the ORFs that are not likely to be coding for a protein. Functional annotations are then sought for each gene using complementary approaches: sequence homology with proteins of known function [stored for instance in UniprotKB (UniProt, 2008)], conservation of genomic structure with annotated species (e.g. synteny), and prediction of functional domains (Apweiler *et al.*, 2000; Claudel-Renard *et al.*, 2003). Combining the above methods and information sources increases the reliability of the annotation transfers from proteins of known function to new genes. Current annotation pipelines succeed at assigning a function to 50–80% of the genes (Serres *et al.*, 2004). A number of databases provide such automatically generated annotations for most sequenced bacterial genomes (see Table 1).

In order to build a metabolic model, it is necessary to identify the specific chemical conversions catalyzed by each enzyme, together with the corresponding stoichiometries. Functional annotations of enzymes therefore need to be translated into appropriate chemical equations. The Enzyme Commission (EC) numbers classification offers an unambiguous way to identify enzyme-catalyzed reactions. When provided by the enzyme annotations, these numbers directly specify which reactions they catalyze. Several enzyme and metabolic databases provide the correspondence between EC numbers and reactions (see Tables 1 and 2). These metabolic databases are often comprehensive catalogues of known biochemical reactions with the associated chemical information, including stoichiometry: they include most of the reference knowledge needed to build metabolic models.

**Table 2:** Type of information provided by each data source

Type of information	
Biochemical activities	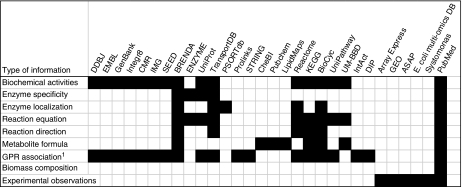
Enzyme specificity	
Enzyme localization	
Reaction equation	
Reaction direction	
Metabolite formula	
GPR association	
Biomass composition	
Experimental observations	

Several issues hinder this translation process. First, enzymatic activities that have been identified only recently are usually not included in the EC classification. Furthermore, full EC numbers are not always systematically assigned in the annotation process. As a result, many annotations retrieved from protein databases are only textual (as in UniProtKB) or ontology based [as in Gene Ontology (Ashburner *et al.*, 2000)] and do not provide the required metabolic information directly. To address this shortcoming, pathologic, the metabolic network reconstruction software tied to the BioCyc metabolic databases, includes an algorithm performing the identification of gene-reaction links from textual annotations (Karp *et al.*, 2002) (see Table 3). This procedure relies on a dictionary of synonyms, however, and may fail at recognizing an explicit reaction when uncommon terms are used. An expert curation step is thus necessary, for which metabolic pathway databases provide useful guidance. Recent initiatives specifically aim at solving this issue: for instance, textual annotations in UniProtKB/SwissProt are being progressively replaced by direct references to reactions from UniPathway, a metabolic database in which all reaction steps are specified up to the chemical level (see Table 1).

**Table 3:** Methods for model reconstruction

**Metabolic model reconstruction (beyond the use of dedicated metabolic databases)**	
Identification of metabolic reactions from textual gene annotations	Karp *et al.* (2002)
Direct inference of metabolic reactions from genome sequence	Sun & Zeng (2004), Arakawa *et al.* (2006), Notebaart *et al.* (2006)
Use of metabolic context to complete pathways	Karp *et al.* (2002), Arakawa *et al.* (2006), DeJongh *et al.* (2007)
**Metabolic model consistency checks**	
Flux variability analysis: identification of reactions that are predicted to never carry any flux	Mahadevan & Schilling (2003)
Identification of dead-end metabolites, which can never be produced or consumed.	Segrè*et al.* (2003), Ebenhöh *et al.* (2004), Imielinski *et al.* (2005), Kumar *et al.* (2007)
Assessment of thermodynamic consistency and assignment of reaction directions.	Yang *et al.* (2005), Kümmel *et al.* (2006a, b)
**Gap filling and model expansion**	
Graph-based metabolic network expansion using shortest metabolic paths	Arita (2003), Boyer & Viari (2003)
GapFill: optimization-based network expansion and reaction reversibility changes to solve dead-end metabolite inconsistencies	Kumar *et al.* (2007)
Optimization-based metabolic network expansion to resolve inconsistent growth phenotypes	Reed *et al.* (2006a, b)
Network-based identification of candidate genes for orphan metabolic activities	Osterman & Overbeek (2003), Green & Karp (2004), Chen & Vitkup (2006), Kharchenko *et al.* (2006), Fuhrer *et al.* (2007)

The broad specificity of some enzymes may also significantly increase the number of distinct reactions they can catalyze. For instance, enzymes annotated with alcohol dehydrogenase activity (EC 1.1.1.1) may catalyze the degradation of several distinct alcohols. Similarly, enzymes acting on lipids are often not specific to the length of their carbon chain. In such cases, functional annotations often report the activity using generic metabolites (e.g. ‘an alcohol’ or ‘a fatty acid’) representing the entire set of possible substrates. Instantiating reactions with specific metabolites is required when building a metabolic model, however, as accounting for the mass balance constraint requires that all metabolites should be well defined. It is thus necessary to identify for each generic compound the corresponding set of specific compounds, as much for primary substrates as for cofactors. This task is complicated by the combinatorial effect, because the number of substrate combinations may significantly increase the number of specific reactions. To address this issue, databases of chemical species can be used to identify all metabolites of a given chemical category (see Tables 1 and 2). In order to determine which metabolites are preferentially recognized by enzymes, processing the literature or browsing information collected in enzyme databases such as BRENDA (Barthelmes *et al.*, 2007) is often necessary. Metabolites involved in metabolic pathways that have already been inferred may also help in selecting the most relevant substrates.

Alternative approaches to metabolic network reconstruction bypass the classical annotation step altogether, taking instead advantage of the curated links between enzyme-encoding gene sequences and reactions [or EC numbers, as in the Genome-Based Modeling (gem) system (Arakawa *et al.*, 2006)] provided by some metabolic databases. Orthology relationships are sought between reference sequences from these databases and the coding sequences from the new genome. While these methods [e.g. autograph (Notebaart *et al.*, 2006), or identics (Sun & Zeng, 2004), see Table 3] simplify the reconstruction process, they usually do not benefit from advanced annotation techniques, such as those derived from structural genomics or domains recognition, and are more difficult to combine with expert annotation. They are also conditioned on the availability of curated gene-reaction associations for a set of reference organisms.

The reconstruction of the metabolism of a new organism can also benefit from the knowledge of complete pathways in related organisms. Metabolic databases often group reactions into pathways or modules that indicate known co-occurrence relationships between reactions that hold across several organisms. Three main resources provide this type of information: MetaCyc (Caspi *et al.*, 2006), KEGG Modules (Kanehisa *et al.*, 2007), and SEED (Overbeek *et al.*, 2005) (see Tables 1 and 2). Metabolic model reconstruction procedures tied to such databases can exploit the known co-occurrences of reactions across reference organisms whose metabolism has been extensively studied (Arakawa *et al.*, 2006). An instance of a reconstruction procedure taking advantage of this notion of metabolic context is again pathologic, which infers the presence of pathways rather than that of single reactions when possible. A reconstruction procedure based on the SEED database was also proposed recently (DeJongh *et al.*, 2007); it includes a check that the inferred pathways can be properly connected to form a ‘working’ model. By leveraging a specific form of ‘guilt-by-association,’ approaches of this type may be able to retrieve reactions catalyzed by enzymes that cannot be correctly identified using current methods. In addition, the presence of spontaneous reactions in the organism may be identified by the occurrence of neighboring reactions in reference metabolic pathways.

In addition to their equations, the reversibility and localization of reactions need to be determined for metabolic models. Few metabolic or enzyme databases report on the reversibility of reactions in *in vivo* conditions (see Table 2). When not found in the literature, reversibility is therefore often determined using simple thermodynamic considerations based on the reaction Gibbs energy, if it is known, or on basic rules depending on the energy equivalents (e.g. NADH or ATP) involved in the reactions (Ma & Zeng, 2003; Kümmel *et al.*, 2006a). Even though very few compartments divide bacterial cells (with periplasm and cytoplasm as the only main compartments in gram-negative bacteria), the presence of such physical separation between metabolites need to be included in their metabolic models. Enzymes present in one compartment cannot interact with metabolites present in another one. To properly model the effect of compartments, the localization of enzymes and the transport of metabolites need to be determined. Information on the localization of enzymes and reactions is seldom included in metabolic databases. Curated versions of BioCyc databases, especially MetaCyc, are a welcome exception, however (Caspi *et al.*, 2006). When not found in the literature, localization can be inferred using *ab initio* predictions from enzyme sequences (Schneider & Fechner, 2004), or determined experimentally, for example using fluorescence microscopy (Meyer & Dworkin, 2007). Transport of metabolites can be inferred using comparative genomics tools that identify transport enzymes [e.g. TransportDB (Ren *et al.*, 2004)]. Yet, such methods hardly determine the specificity of transporters; knowledge of transported metabolites is therefore often completed using direct information on the microorganism's physiology and the metabolites it was shown to utilize in growth experiments.

Overall, reconstructing a constraint-based model for an organism's metabolism involves collecting various types of information. A summary of the respective contributions of each data source to the model construction is shown in Table 2.

### Checking the consistency of reconstructed models

**Fig. 2:**
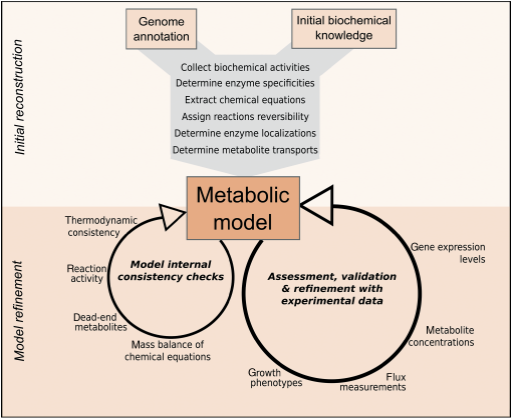
Pipeline for model reconstruction and refinement. An initial model is reconstructed from genome annotations and from preexisting knowledge on the species' biochemistry and physiology. Besides collecting the biochemical activities, this task includes several additional key steps. The resulting model is then iteratively corrected and refined, according to internal consistency criteria and by comparing its predictions to experimental data.

Once a draft metabolic model is obtained, its consistency can be checked using a set of simple tests (see Fig. 2): is the model chemically and physically coherent? Are there remaining ‘dead-ends’ in metabolic pathways or reactions bound to be inactive? Is the model able to produce essential metabolites from a known growth medium?

Constraint-based metabolic models fundamentally rely on reaction stoichiometries to properly account for the mass balance in metabolism at steady state. It is therefore crucial that all chemical equations are correctly balanced to avoid unrealistic creation or destruction of matter. To control the correctness of the reaction stoichiometries, the atom balance of each reaction can be checked using the chemical formulae of the metabolites, which are typically found in databases of chemical compounds (see Table 1). For cases where the formula is not available for all metabolites, a method was recently introduced to detect such balance errors in metabolic models by solely comparing chemical equations – for instance, reactions A→B and A→B+C would be identified by this method as ‘stoichiometrically inconsistent,’ because balancing both equations would require that at least one of the metabolites has a null or negative mass (Gevorgyan *et al.*, 2008).

The assumptions on which constraint-based models are founded do not enforce thermodynamic consistency on the fluxes. Flux distributions obeying conservation of mass can still include internal cycles that violate thermodynamic laws, allowing for instance the artificial generation of high-energy cofactors. To prevent models from predicting such unrealistic metabolic modes, extensions of the modeling framework were proposed that directly enforce these laws (Beard *et al.*, 2002). Their nonlinear nature entails costly computations, however, which hinder the use of such modeling extensions in practice. In order to provide thermodynamically consistent models without including such extensions, methods have been developed to detect inconsistent cyclic modes in draft metabolic models, and propose changes in reaction reversibility that would avoid those modes from being predicted (Yang *et al.*, 2005; Kümmel *et al.*, 2006a).

Before one can reap the benefits of having a model, the model should be functional, i.e. it should be checked that non-null fluxes can actually be predicted. This relates to the completeness of the model, because for instance a missing reaction in a linear pathway would prevent any non-null flux from being predicted in it at steady state, thereby inactivating all other reactions in the pathway. Metabolites that are never consumed or never produced, so-called ‘dead-ends,’ typically reveal that reactions are missing. In order to help investigate and correct these so-called ‘metabolic gaps,’ methods have been developed that assess whether reactions can be active in the model (Reed & Palsson, 2004), identify dead-end metabolites (Kumar *et al.*, 2007) or directly track the producibility of metabolites from source metabolites (Segrè*et al.*, 2003; Ebenhöh *et al.*, 2004). In case the model is later used to predict growth phenotypes (see Applications of metabolic models), the producibility of biomass precursors and the completeness of their biosynthetic pathways should be especially checked beforehand. Dedicated procedures have been designed to systematically perform these checks for newly reconstructed models (Segrè*et al.*, 2003; Imielinski *et al.*, 2005; Senger & Papoutsakis, 2008). Solving such inconsistencies often involves filling metabolic gaps or completing the network with additional metabolic pathways.

The methods presented in this section check the consistency of the reconstructed model with respect to a set of basic rules (see Table 3). We will review in the section on model applications how model predictions can also be confronted with experimental data, providing consistency checks of the model with respect to diverse additional experimental evidence. Interpreting and solving identified inconsistencies of either type are key to improving the quality of the metabolic model.

### Targeted searches for missing metabolic activities

Consistency checks (either internal to the model or relative to experimental datasets) may show that the reconstructed model is incomplete and lacks some metabolic reactions. Resolving these metabolic gaps entails expanding the model by identifying and including missing biochemical activities. This process basically consists of two steps: (1) identifying plausible candidate reactions that could complete the model and (2) finding genes that could catalyze the hypothesized activities.

Reactions contained in metabolic databases are the primary source of information for completing the metabolic model (see Table 1). The search for candidate reactions within these databases can be facilitated using knowledge of existing pathways (as in MetaCyc, SEED, or UM-BBD, see Table 1) or computational methods (Arita, 2003; Boyer & Viari, 2003; Kumar *et al.*, 2007) (see Table 3). In the latter category, the GapFill method was specifically developed to identify dead-ends in models, and correct them by adding reaction from a global repository of reactions, changing the reversibility status of reactions, or adding transporters (Kumar *et al.*, 2007). The addition of reactions to the model is guided by an optimization step minimizing the number of reactions. Similarly, Reed *et al.* (2006b) proposed a method which drives the expansion of the metabolic model to account for the utilization of additional external compounds. For metabolites experimentally shown to be used by the organism but not predicted as such by the model (see Applications of metabolic models on growth phenotype predictions for methods to perform these predictions), their method automatically proposes minimal sets of reactions from a repository of reactions that, if added, would allow the model to exploit the external metabolites.

The set of reactions referenced in metabolic databases is far from being comprehensive: the right candidates for completing the model may not yet be known. Computational and experimental approaches have been proposed to extend this ‘universe of possible reactions.’ On the computational side, several methods originating from the field of chemo-informatics have been designed to infer chemical transformations (Gasteiger, 2005). Some of them have been more specifically adapted to biochemical transformations, using rules on enzymatic conversions to infer new conversions for biologically relevant metabolites (Klopman *et al.*, 1994; Arita, 2000; Hatzimanikatis *et al.*, 2005; Ellis *et al.*, 2008).

Numerous experimental methods are also being developed to explore the range of possible biochemical reactions. MS and nuclear magnetic resonance (NMR) techniques are able to identify and quantify large sets of metabolites at high throughput (Dunn *et al.*, 2005; Dettmer *et al.*, 2007). Computational methods have been proposed to infer reactions from MS data, by analyzing mass differences between related metabolites (Breitling *et al.*, 2006) or correlations between metabolite concentrations across distinct conditions (Steuer, 2006). They do not provide direct evidence for biochemical transformations, however: their predictions should be treated as clues to be confirmed by additional information. Although mostly used to determine metabolic fluxes, atom-labeling experiments could also become powerful tools to elucidate novel metabolic pathways (Sauer, 2006). They can advantageously complement computational *ab initio* pathway inference methods by selecting candidate pathways that are compatible with observed isotopic patterns. Finally, untargeted enzyme activity screenings have recently been performed to identify the substrates of enzymes of unknown function and discover novel activities (Saghatelian *et al.*, 2004; Saito *et al.*, 2006). The availability of large-scale libraries of ORF clones (Kitagawa *et al.*, 2005) should increase the likelihood of such methods expanding the store of known reactions.

The search for candidate genes for orphan metabolic activities is in some ways the reverse of the classical genome annotation problem (i.e. searching the function of identified genes). Yet, many of the tools developed to determine gene functions can be adapted for this purpose. Sequence homology to already characterized genes is central to most methods for candidate gene detection, but combining it with additional types of evidence can significantly improve performance. For instance, several approaches exploit functional links, such as gene neighborhood, gene co-expression, protein interaction, or phylogenetic co-occurrence, to relate candidate genes with genes involved in the same metabolic pathways or close in the metabolic network (Osterman & Overbeek, 2003; Green & Karp, 2004; Chen & Vitkup, 2006; Kharchenko *et al.*, 2006; Fuhrer *et al.*, 2007). Databases such as STRING (von Mering *et al.*, 2007) or Prolinks (Bowers *et al.*, 2004) compile large sets of functional links across a wide range of organisms. On the experimental side, enzyme activity screenings are used to validate the generated candidates. Furthermore, when the orphan activity is associated to a specific phenotype, screens of systematic knockout mutant phenotypes can help in identifying candidates (Aghaie *et al.*, 2008).

The two types of methods – finding candidate reactions or candidate genes – benefit from being used in combination, as identifying genes for putative reactions can help in selecting the proper reactions to include.

## Applications of metabolic models

A wealth of computational methods has been developed to help analyze biological properties revealed by reconstructed metabolic models. Not only would a comprehensive and technical description exceed the scope of this review, but these methods have been extensively covered elsewhere, either on the technical side (Price *et al.*, 2004) or for applications on a specific organism, i.e. *E. coli* (Feist & Palsson, 2008). We will provide here the reader with a review on the main applications for which constraint-based models have been most successful and are mostly promising for bacterial species. We will distinguish four main types of applications: (1) analysis of network properties of metabolism, (2) prediction and analysis of bacterial growth phenotypes, (3) model-based interpretation of experimental data, and (4) metabolic engineering.

### Analysis of network properties

The principle of constraint-based modeling consists in studying the set of reaction fluxes – namely flux distributions – that are achievable at steady state given the constraints imposed on the system. Reaction fluxes can vary inside a continuous set of possible values. This set can encompass significant variability at the level of individual pathway or reaction fluxes. A wide range of methods have been designed to explore that variability and analyze specific properties of metabolites and reactions which emerge from the flux constraints.

One approach consists in sampling the set of achievable flux distributions (Almaas *et al.*, 2004; Reed & Palsson, 2004; Wiback *et al.*, 2004). Methods that provide a uniform sampling of the possible states have been proposed (Almaas *et al.*, 2004; Wiback *et al.*, 2004). By sampling a significant number of metabolic states, these approaches offer an overview of the range of flux distributions that can occur in the metabolic network at steady state. The ‘uniform’ nature of the sampling is based only on the mathematical description of the set of possible flux distributions, avoiding any prior assumption on which metabolic states are most likely to be selected *in vivo*. For instance, these sampling methods have been used to evaluate the relative occurrence of reactions within the set of possible flux distributions and across several environmental conditions (Almaas *et al.*, 2004). This analysis showed that a few reactions are active in many sampled flux distributions and carry high fluxes – forming a so-called high-flux metabolic backbone – while many others are active in few sampled flux distributions and carry low fluxes. Similar methods were also used to evaluate the correlation of flux values between pairs of reactions across sampled metabolic states (Reed & Palsson, 2004; Becker *et al.*, 2007) and thereby determine metabolic dependencies between reactions. From a more theoretical angle, sampling was also used to evaluate the size of the set of possible flux distributions (Wiback *et al.*, 2004; Braunstein *et al.*, 2008). When computed for distinct (genetic perturbation × environmental condition) pairs, the relative sizes of the corresponding flux distribution sets were interpreted as indicators of the respective diversity of metabolic states in the tested conditions (Wiback *et al.*, 2004).

The diversity of achievable metabolic fluxes can also be evaluated locally for each reaction. Flux variability analysis was designed for this purpose: an optimization procedure computes the minimal and maximal allowed flux of each reaction independently (Mahadevan & Schilling, 2003). This procedure identifies reactions that do not carry any flux, or conversely those that carry non-null flux in all possible metabolic states. Flux variability analysis has been broadly used to predict the activity of reactions for specific sets of metabolic constraints (Mahadevan & Schilling, 2003; Reed & Palsson, 2004; Teusink *et al.*, 2006; Feist *et al.*, 2007; Henry *et al.*, 2007; Shlomi *et al.*, 2007a).

Flux sampling or flux variability approaches only provide partial description of the set of possible flux distributions. To get a comprehensive picture of the possibilities, methods which compute elementary modes (Schuster *et al.*, 2000) and extreme pathways (Schilling *et al.*, 2000) have been developed. These notions differ only slightly in their mathematical formulation (Klamt & Stelling, 2003; Papin *et al.*, 2004): the main idea is to determine the set of elementary and independent metabolic routes that can occur in the metabolic model. These elementary routes are flux distributions that (1) respect all assumed constraints, including steady state and irreversibility, and (2) are elementary in the sense that they are composed of a minimal set of active reactions. This second condition ensures that the flux distribution is not decomposable into a combination of smaller elementary routes. It can be shown that any achievable flux distribution can be expressed as a combination of such elementary routes. This property, together with the fact that the set of elementary routes is unique, independently of the method used to compute it (Klamt & Stelling, 2003), has inspired numerous applications. This subfield is also known as metabolic pathway analysis. For instance, elementary modes and extreme pathways have been used to exhaustively describe the independent metabolic routes occurring in newly reconstructed models, often sorted by metabolic function (Schilling & Palsson, 2000; Van Dien & Lidstrom, 2002; Papin *et al.*, 2002). The redundancy of routes can be assessed and the respective yields of routes of conversion can be compared (Papin *et al.*, 2002). Conversely, the relative importance of reactions in metabolism was scored using elementary routes, reactions involved in many routes being likely to be key players in metabolism (Stelling *et al.*, 2002). Finally, metabolic dependencies between reactions which are stronger than those determined only by analyzing the correlation of fluxes in sampled distributions can be deduced from knowing elementary routes. Reactions that always appear jointly in elementary routes are bound to operate together (Pfeiffer *et al.*, 1999). The main obstacle in metabolic pathway analysis is the size and complexity of the metabolic models, as the number of elementary routes dramatically increases with the size of the model (Yeung *et al.*, 2007). The computation of all routes is currently only tractable for medium-size models, although significant progresses have been made recently (Terzer & Stelling, 2008).

Alternative approaches have been developed in order to explore metabolic dependencies in models of larger size. One of them, flux coupling analysis, has become a popular analytical tool (Burgard *et al.*, 2004). Flux coupling analysis identifies all pairs of reactions whose fluxes are always coupled at steady state. It has been used in a wide range of studies, and the resulting sets of coupled reactions were for instance compared with correlations observed in the transcriptional states of enzymes (Reed & Palsson, 2004; Notebaart *et al.*, 2008), interpreted with respect to the structure of the metabolic regulation (Notebaart *et al.*, 2008), and used to study the horizontal transfer of genes during bacterial evolution (Pál *et al.*, 2005a, b). Similar methods were developed to study metabolic relationships between metabolites, either by simply examining the co-occurrence of metabolites in reactions (Becker *et al.*, 2006) or by determining conservation relations between metabolites (Nikolaev *et al.*, 2005; Imielinski *et al.*, 2006). This last type of method was applied to determine coupling relationships between metabolite concentrations, identify metabolite pools sharing conserved chemical moieties (Nikolaev *et al.*, 2005), and exhaustively predict distinct minimal growth media for *E. coli* (Imielinski *et al.*, 2006).

### Prediction of growth phenotypes

One of the primary uses of genome-scale metabolic models is the prediction of growth phenotypes (Price *et al.*, 2004; Palsson, 2006). Because these models aim at comprehensiveness, they are able to account for all main metabolic processes contributing to growth, i.e. the production of energy and biomass precursors from external metabolites. Growth phenotypes can therefore be predicted by examining to which extent metabolic requirements for growth, in terms of energy generation and biomass precursors synthesis, can be fulfilled by flux distributions from the model. Growth phenotypes can be predicted either in a qualitative manner (prediction of the mere ability to grow) by checking piecemeal for the producibility of each biomass precursor metabolite (Imielinski *et al.*, 2005), or in a quantitative manner (prediction of growth performance) by including a biomass reaction consuming them in proportion to their ratio in biomass composition and studying the flux values it can attain (Price *et al.*, 2004). Determining biomass composition is therefore a necessary prerequisite to growth phenotype predictions. This is often achieved by examining the relevant literature or adapting known biomass compositions of related organisms. The Flux Balance Analysis (FBA) method was specifically designed to predict quantitative growth phenotypes (Varma & Palsson, 1994b; Price *et al.*, 2004). It computes the maximal growth yield achievable in the metabolic model by maximizing the biomass reaction flux (representing the growth rate) given a set of bounded intake rates for external substrates. FBA relies on the strong assumption that bacteria have optimized their growth performance in a subset of possible environments during their evolution, thereby making the maximization of biomass production a driving principle for metabolic operation (Varma & Palsson, 1994b). This assumption has been confirmed by experiments in several cases (Edwards *et al.*, 2001). Using FBA, global quantitative relationships can be predicted between the input rates of nutrients, the output rates of byproducts, and the growth rate (Stephanopoulos *et al.*, 1998; Edwards *et al.*, 2002; Price *et al.*, 2004).

The global energy consumption of the cell can significantly influence the outcome of quantitative growth phenotype predictions. Two ATP hydrolysis fluxes are added to the models in order to properly account for it. One is constant and models the non-growth-associated maintenance, which represents the fraction of the energy demand necessary for the cell survival that is independent from its growth rate, for example to maintain the right ionic strength (Stouthamer & Bettenhaussen, 1973). The second flux is proportional to the growth rate and corresponds to the energy demand associated with growth beyond the mere requirements of metabolic pathways – which are already directly accounted for in the model – for example energy for cell division or assembly of higher order cell structures. These two parameters are usually determined by fitting growth yield predictions derived using FBA to measured growth yields provided by growth monitoring experiments (Reed *et al.*, 2006a). Measurements of growth yields for distinct growth rates are sufficient to fit both growth-associated and non-growth-associated maintenance parameters (Varma & Palsson, 1994a). The values of these parameters were determined using experimental growth measurements for a significant proportion of reconstructed models (see Table 4).

**Table 4:** Existing genome-scale metabolic models for bacterial organisms

					Experimental assessment		
Organism	Reference	Genes	Reactions*	Metabolites†	Wild-type growth phenotypes	Knockout mutant growth phenotypes	Quantitative growth measures
*Acinetobacter baylyi*	Durot *et al.* (2008)	774	875	701	173/190 (91%)	1138/1208 (94%)	–
*Bacillus subtilis*	Oh *et al.* (2007)	844	1020	988	200/271 (74%)	720/766 (94%)	–
*Clostridium acetobutylicum*	Lee *et al.* (2008a)	432	502	479	10/11 (91%)	–	X
*Clostridium acetobutylicum*	Senger & Papoutsakis (2008)	474	552	422	–	–	–
*Escherichia coli*‡	Feist *et al.* (2007)	1260	2077	1039	129/170 (74%)	1152/1260 (92%)	X
*Geobacter sulfurreducens*	Mahadevan *et al.* (2006)	588	523	541	–	–	X
*Haemophilus influenza*	Schilling & Palsson (2000)	412	461	367	–	–	–
*Helicobacter pylori*§	Thiele *et al.* (2005)	341	476	485	–	54/72 (75%)	–
*Lactobacillus plantarum*	Teusink *et al.* (2006)	721	643	531	–	–	X
*Lactococcus lactis*	Oliveira *et al.* (2005)	358	621	422	–	–	X
*Mannheimia succiniciproducens*	Hong *et al.* (2004)	335	373	332	–	–	–
*Mycobacterium tuberculosis*	Beste *et al.* (2007)	726	849	739	–	547/705 (78%)	X
*Mycobacterium tuberculosis*	Jamshidi & Palsson (2007)	661	939	828	–	132/237 (56%)	X
*Neisseria meningitidis*	Baart *et al.* (2007)	555	496	471	–	–	X
*Pseudomonas aeruginosa*	Oberhardt *et al.* (2008)	1056	883	760	78/95 (82%)	893/1056 (85%)	–
*Pseudomonas putida*	Nogales *et al.* (2008)	746	950	710	84/90 (93%)	665/746 (89%)¶	X
*Rhizobium etli*	Resendis-Antonio *et al.* (2007)	363	387	371	–	–	–
*Staphylococcus aureus*	Becker & Palsson (2005)	619	641	571	–	–	–
*Staphylococcus aureus*	Heinemann *et al.* (2005)	551	774	712	–	8/14 (57%)	–
*Streptomyces coelicolor*	Borodina *et al.* (2005)	700	700	500	54/58 (93%)	11/12 (92%)	X

First two columns of experimental assessment show the number of correct predictions among all experimentally determined qualitative growth phenotypes. Last column specifies whether the model has been assessed against quantitative growth rate measurements.

* Number of distinct reactions including transport processes.

† Number of biochemically distinct metabolites.

‡ This model is an update of two earlier models for *E. coli* (Edwards & Palsson, 2000; Reed *et al.*, 2003).

§ This model is an update of an earlier model for *H. pylori* (Schilling *et al.*, 2002).

¶ Using gene essentiality data for *Pseudomonas aeruginosa*.

Once fitted, and assuming these parameters remain constant across environments, the model can be used to predict growth rates on different media (Edwards *et al.*, 2001). Predicted growth yields revealed to be consistent with observed ones on a significant number of media for *E. coli* (Edwards *et al.*, 2001). Inconsistencies between predicted and observed growth yields can have multiple interpretations. First, the assumption of optimal substrate utilization can be questionable for growth predictions on environments that are not commonly encountered by the organism (Ibarra *et al.*, 2002; Schuster *et al.*, 2008). Using an adaptive evolution experiment on *E. coli* cells grown in glycerol minimal medium, Ibarra and colleagues actually observed that, while the initial growth yield was suboptimal, it progressively evolved to reach the optimal value predicted by the model. Other biological constraints, such as regulation or capacity constraints, may also prevent the organism from using optimal flux distributions (Oliveira *et al.*, 2005; Feist *et al.*, 2007). Comparing predictions of growth phenotypes with experimental measures may also help in refining the model. A model component that is often refined using quantitative growth predictions is the stoichiometry of proton translocation that occurs in reactions of electron transport systems, such as the respiratory chain. These stoichiometries are often hard to determine *a priori*, yet they impact directly the P/O ratio and the efficiency of energy generation (Reed *et al.*, 2006a). With the help of a metabolic model and growth yield measurements on several distinct media, Feist *et al.* (2006) studied the unknown proton translocation stoichiometry of such a reaction in *Methanosarcina barkeri* by determining for each media the model maintenance parameters that provided the best growth yield predictions for different hypothesized values of the stoichiometry. Assuming that maintenance should not significantly change across media, they selected the stoichiometry that triggered the smallest variation among the determined maintenance parameters across the environments. Other studies investigated the stoichiometry of proton translocation in the respiratory chain by directly exploiting measured ratios of electron acceptor (e.g. oxygen, or Fe(III) in *Geobacter sulfurreducens*) consumption rate vs. carbon source consumption rate and growth rate (Heinemann *et al.*, 2005; Mahadevan *et al.*, 2006).

Models can readily predict the effect of gene deletion on growth phenotypes. To that end, a layer of Gene Protein Reaction associations – usually called GPR (Reed *et al.*, 2003) – is added to the model to predict the effect of gene deletion on reaction activity. Each reaction is associated to its enzyme-encoding genes by a Boolean rule: genes encoding for subunits of an enzymatic complex are linked with an AND rule, while genes encoding for alternative enzymes are linked with an OR rule. Using GPR rules, gene deletions are translated into ‘blocked’ reactions, which are then inactivated in the model by constraining their fluxes to zero. FBA can be applied to predict growth phenotypes of gene knockout mutants. Nevertheless, the hypothesis of optimal growth is largely debatable for such genetically engineered mutants, as their metabolism was not exposed to evolutionary pressure. Basing on the assumption that metabolism in a knockout mutant operates as closely as possible to metabolism in the wild-type strain, two specific methods were introduced. They predict knockout mutant growth phenotypes by minimizing either the overall flux change [MoMA (Segrè*et al.*, 2002)] or the number of regulatory changes [ROOM (Shlomi *et al.*, 2005)] between the wild-type strain and the mutant strain (see Table 5). Both methods were shown to provide slightly better predictions than FBA.

**Table 5:** Main analytical methods for genome-scale models sorted by type of application

**Analysis of network properties**	
Flux sampling: random sampling of flux distribution among the set of possible metabolic states	Almaas *et al.* (2004), Reed & Palsson (2004), Wiback *et al.* (2004)
Flux variability analysis: examination of flux variability for each reaction	Mahadevan & Schilling (2003)
Metabolic pathway analysis, elementary modes/extreme pathways: comprehensive description of all independent metabolic modes achievable in the metabolic network	Schilling *et al.* (2000), Schuster *et al.* (2000), Klamt & Stelling (2003)
Flux coupling: identification of reaction pairs whose fluxes are coupled	Burgard *et al.* (2004)
Metabolite coupling/evaluation of conserved metabolite pools	Nikolaev *et al.* (2005), Becker *et al.* (2006), Imielinski *et al.* (2006)
**Prediction and interpretation of bacterial growth phenotypes**	
Producibility analysis of biomass precursors	Imielinski *et al.* (2005)
FBA: quantitative prediction of growth yield by maximization of growth rate given bounded nutrient input rates	Varma & Palsson (1994a, b)
MOMA: prediction of gene deletion mutant flux distribution by minimizing overall flux changes with wild type	Segrè*et al.* (2002)
ROOM: prediction of gene deletion mutant growth by minimizing regulatory changes with wild type	Shlomi *et al.* (2005)
Identification of multiple gene deletion essentialities	Klamt & Gilles (2004), Deutscher *et al.* (2006), Imielinski & Belta (2008)
**Model-based interpretation of experimental data**	
*Metabolic flux measurements*	
Metabolic Flux Analysis using labeled metabolites: prediction of attainable reaction fluxes given observed metabolite isotopic patterns	Wiechert (2001), Sauer (2006)
Global prediction of reaction activities using metabolic flux measurements on subsets of reactions	Herrgård *et al.* (2006a, b)
Identification of metabolic objectives best describing observed fluxes	Burgard & Maranas (2003), Schuetz *et al.* (2007)
*Metabolite concentrations*	
Comparison of model coverage with experimentally detected metabolites	Oh *et al.* (2007)
NET analysis and TMFA: application of thermodynamic constraints to reaction directions using metabolite concentrations	Kümmel *et al.* (2006a, b), Henry *et al.* (2007)
*Gene expression*	
Identification of metabolic pathways correlated with gene expression levels	Schwartz *et al.* (2007)
Refinement of flux distribution predictions by blocking reactions corresponding to unexpressed genes	Akesson *et al.* (2004)
Evaluation of consistency of gene expression levels with metabolic objectives	Becker & Palsson (2008)
rFBA and SR-FBA: prediction of gene expression states using Boolean regulatory rules	Covert *et al.* (2001), Barrett *et al.* (2005), Barrett & Palsson (2006), Shlomi *et al.* (2007a, b)
**Metabolic engineering**	
Systematic identification of gene deletions enhancing metabolite production yield	Burgard *et al.* (2003), Patil *et al.* (2004), Alper *et al.* (2005a, b)
OptStrain: systematic identification of reaction additions enabling the production of novel metabolites	Pharkya *et al.* (2004)
Prediction of adjustments of enzyme expression levels enhancing metabolite production yield	Pharkya & Maranas (2006), Lee *et al.* (2007)

The throughput of experiments evaluating qualitative growth phenotypes – i.e. described simply as *viable* or *lethal*– has increased dramatically in the last few years. Phenotype Microarrays from Biolog Inc. typically report growth phenotypes for several hundreds of media in a single experiment (Bochner *et al.*, 2001). In parallel to this, collections of knockout mutants are being built for a growing number of bacteria (Akerley *et al.*, 2002; Jacobs *et al.*, 2003; Kobayashi *et al.*, 2003; Baba *et al.*, 2006; Liberati *et al.*, 2006; Suzuki *et al.*, 2006; de Berardinis *et al.*, 2008). The systematic assessment of growth phenotypes of knockout mutants provides a significant resource for exploring the metabolic capabilities of organisms and investigating their gene functions (Carpenter & Sabatini, 2004), but their direct interpretation is made difficult by the complexity and size of metabolic networks (Gerdes *et al.*, 2006). These results can be readily compared with model predictions, however, providing a way to interpret them and assess the model correctness. Given the qualitative nature of these growth phenotypes, two types of inconsistencies may arise: *false viable* predictions – growth was predicted yet not observed experimentally – and *false lethal* predictions – growth was not predicted yet observed experimentally. On the one hand, these inconsistencies may be caused by limitations of the model or cases where the modeling assumptions do not hold. Regulation may for instance trigger a lethal phenotype by blocking an alternate pathway, which would not be predicted as blocked in the merely metabolic model. On the other hand, examining the inconsistencies may identify errors in the model and lead to its refinement. All model components may comprise errors, including the GPR associations, the metabolic network itself, and the stated biomass requirements. False lethal predictions are often clues that some biomass component is actually not essential, or that the model lacks an alternative gene or pathway that would allow it to survive in the given experimental conditions. Conversely, false viable predictions can help detect missing essential biomass components, genes falsely annotated as encoding isozymes or reactions that were wrongly assigned or are inactive in the experimental conditions (Duarte *et al.*, 2004; Joyce *et al.*, 2006). Growth phenotype predictions have been evaluated for a significant proportion of reconstructed models, whenever experimental data were available (see Table 4). Interpretation of inconsistent cases by expert examination led to several annotation and model refinements, some of which were supported by the results of targeted experiments (Covert *et al.*, 2004; Duarte *et al.*, 2004; Joyce *et al.*, 2006; Reed *et al.*, 2006b). Automated methods were recently introduced to systematically look for interpretations of inconsistencies and possible modifications in the model. Corrections of the GPR associations can be systematically proposed that match the gene essentiality observation with predicted reaction essentiality (M. Durot *et al.*, unpublished data). With regard to the metabolic network itself, metabolic gap filling approaches have been adapted to propose network corrections that resolve wrongly predicted growth phenotypes (Reed *et al.*, 2006b). Finally, valuable insights into the determination of essential biomass precursors can be provided by methods that analyze correlations between lethality and metabolite production (Imielinski *et al.*, 2005; Kim *et al.*, 2007). All these methods act independently on distinct components of the model. A unifying method integrating all types of corrections, which is yet to come, could lead to an integrated platform for the systematic interpretation of upcoming growth phenotyping results.

Models can actually predict growth phenotypes for any environmental condition and any combination of gene deletions, which is beyond reach of experiments. Given the combinatorial complexity of mixing several gene deletions, dedicated methods have been designed to analyze the effects of multiple deletions and applied to identify epistatic interactions between genes (Klamt & Gilles, 2004; Deutscher *et al.*, 2006, 2008; Imielinski & Belta, 2008). Prediction of growth phenotypes have also been used to automatically assign condition-dependent roles to genes (Shlomi *et al.*, 2007b), investigate the causes of gene dispensability (Papp *et al.*, 2004; Kuepfer *et al.*, 2005), or study bacterial evolution (Pál *et al.*, 2005a, 2006). These two latter studies on bacterial evolution used an *E. coli* model to analyze the effect of changing growth environments on the acquisition of new metabolic capabilities by horizontal gene transfer (Pál *et al.*, 2005a) and to simulate the reductive evolution of metabolism in specific environmental conditions (Pál *et al.*, 2006).

### Model-based interpretation of experimental data

The recent development of experimental techniques has enabled measurements at genome-scale of several types of quantities, generating so-called ‘omics’ datasets. These datasets provide partial yet comprehensive snapshots of cellular mechanisms (Ishii *et al.*, 2007a), but their interpretation is made difficult by the volume of data. Computational methods are thus needed if meaningful biological results are to be extracted (Joyce & Palsson, 2006). A variety of methods have been developed to exploit experimental data related to metabolic states, for example measurements of metabolic fluxes, metabolite concentrations, enzyme levels, or gene expression, in the light of genome-scale models. Two cases generally arise: either experimental observations are directly comparable to model predictions, or these observations lead to the imposition of additional constraints that refine the set of predicted metabolic states. Observations falling in the second category allow for instance the selection of those metabolic routes that are compatible with the experimental observations, or help predict quantitative values for the fluxes. When directly comparable to model predictions, experimental data may be used to assess model correctness and assumptions, identify inconsistencies, and target improvements, as illustrated above with growth phenotypes (Reed *et al.*, 2006b). We will review such integration methods in the following sections for three types of experimental data: measurement of (1) reaction fluxes, (2) metabolite concentrations, and (3) gene expression levels.

Refining the model with experimental data increases its correctness with respect to the observations but may decrease its predictive power. Predictions performed with a refined model should actually be interpreted with care to avoid circular reasoning: data that have been directly used to improve the model can no more be considered as predictions, they are part of the evidences on which the model is based to perform predictions. For instance, a model whose maintenance parameters have been determined using growth rate measurement can no more *predict* the growth rate for the environmental condition. This problem can become serious when models are extensively fitted with experimental data, as they then become more descriptive than predictive. Nevertheless, some refinement processes applied to genome-scale models involve finding additional biological evidence that supports the refinement, thereby breaking the circular reasoning. For instance, corrections of inconsistent growth phenotype predictions by additions of alternate enzymes often involve finding additional proofs that the introduced enzymes possess the right activity.

#### Metabolic flux measurements

One of the most direct experimental accesses to metabolic fluxes is provided by atom-labeling experiments (Wiechert, 2001; Sauer, 2006). By analyzing the fate of labeled metabolites, valuable information can be deduced about the reactions that are actually taking place. The most common technique for this consists in analyzing the stable isotope patterns (mostly using ^13^C) found in products of metabolism given known isotope patterns in nutrient metabolites (Wiechert, 2001; Sauer, 2006). These data can be properly interpreted only using a metabolic model that includes information about atom mappings for each reaction (Zupke & Stephanopoulos, 1994; Wiechert *et al.*, 1999; Antoniewicz *et al.*, 2007a). Such models have been built for a few organisms, often using existing constraint-based models as a basis (Antoniewicz *et al.*, 2007b; Suthers *et al.*, 2007). While atom mappings for reactions are currently mostly inferred using chemoinformatics methods (Raymond *et al.*, 2002; Arita, 2003; Hattori *et al.*, 2003), this information will likely be made accessible in dedicated databases in the coming years.

By qualitatively examining isotope patterns in nutrients and products, information can already be extracted about the possible routes of conversion (van Winden *et al.*, 2001; Sauer, 2006; Kuchel & Philp, 2008). Patterns in products actually depend on their biosynthetic pathways. Observed patterns that are inconsistent with the predicted possible patterns are clues that other pathways may occur *in vivo*. This approach was for instance recently used to evaluate the model of *G. sulfurreducens*: an inconsistent isotope pattern for isoleucine led to the discovery of an isoleucine biosynthesis pathway previously uncharacterized in this bacteria (Risso *et al.*, 2008).

Quantitative interpretation of isotope patterns together with measurement of extracellular metabolite fluxes can help determine the value of intracellular reaction fluxes using Metabolic Flux Analysis (Zupke & Stephanopoulos, 1994; Stephanopoulos *et al.*, 1998; Wiechert *et al.*, 1999; Sauer, 2006; Antoniewicz *et al.*, 2007a). Known flux values can then be directly exploited in models to characterize which metabolic pathways are operating and quantify their fluxes. As an application, Herrgård *et al.* (2006a) introduced the optimal metabolic network identification method, which combines flux measurements for a fraction of the reactions with the assumption of optimal growth from FBA to globally infer which reactions are active. This method has been for instance used to identify bottleneck reactions that limit the growth in engineered strains, and discard putative reactions from newly reconstructed models (Herrgård *et al.*, 2006a).

Observed fluxes were also used to determine relevant objective functions to choose when predicting metabolic states with FBA (Burgard & Maranas, 2003). By evaluating the match of predicted fluxes with observed ones, these studies could identify those metabolic objectives that provided the best fit. Distinct objectives, including maximization of ATP or biomass yields, were identified for instance in *E. coli* depending on the environmental conditions (Schuetz *et al.*, 2007). Observed metabolic fluxes, however, often show that metabolism does not necessarily operate according to optimality principles (Fischer *et al.*, 2004), especially when regulatory constraints are overlooked.

#### Metabolite concentrations

High-throughput measurement of intracellular metabolite concentrations is becoming common practice thanks to recent developments in MS and NMR technologies (Dunn *et al.*, 2005; Dettmer *et al.*, 2007). Metabolite profiling experiments commonly detect thousands of peaks, among which hundreds can usually be exploited to identify metabolites and determine their concentrations, using for instance known spectra of reference metabolites (Dunn *et al.*, 2005). These datasets, while not fully comprehensive, provide significant information on metabolites present in the cell.

Merely comparing the set of detected metabolites to the set of metabolites present in the model already help in assessing the comprehensiveness of the model. For example, in the reconstruction process of *Bacillus subtilis* metabolic model, Oh *et al.* (2007) evaluated the overlap between model metabolites and intracellular metabolites identified in a metabolomics dataset; among 350 intracellular metabolites identified, only 160 were present in the model. No previously known biochemical activities could be associated with the remaining metabolites, illustrating the fact that a large part of *B. subtilis* metabolism remains unknown. These unaccounted metabolites can guide further investigations on missing activities, leading to expansion of the model's metabolite scope consequently.

By extending the constraint-based modeling framework to encompass thermodynamic constraints on Gibbs energies of reactions, knowledge of absolute metabolite concentrations can be translated into constraints on flux directions (Kümmel *et al.*, 2006b; Henry *et al.*, 2007). A first application is to check the consistency of metabolomic datasets with respect to metabolic fluxes predicted by the model. Methods and software have been developed to pinpoint inconsistent concentration measures (Zamboni *et al.*, 2008). Conversely, metabolomic-derived constraints refine the characterization of metabolic fluxes within the model; their integration has allowed the prediction of ranges of concentrations for unmeasured metabolites, reaction directions, and ranges of Gibbs energies of reactions, identifying thereby potentially regulated reactions (Kümmel *et al.*, 2006b).

Thermodynamic constraints merely enforce link between the concentrations of metabolites and the directions of reactions. Taking reaction kinetics into consideration could reinforce that link and make it more quantitative. Extending models to handle kinetics is still an open issue (Famili *et al.*, 2005; Yugi *et al.*, 2005; Ishii *et al.*, 2007b; Smallbone *et al.*, 2007; Covert *et al.*, 2008; Jamshidi & Palsson, 2008), all the more challenging because of the potential influence of regulation, the scarcity of kinetic parameter values and the lack of scalable analytical methods.

#### Gene expression data

Thanks to technological advances, gene expression levels are among the most widely accessible type of ‘large-scale’ experimental data. While such datasets provide a global overview of the level of expression of enzymes, deriving information on reaction fluxes from gene expression levels is hindered by the numerous biological processes intervening between them. Changes in rates of translation or mRNA and enzyme degradation may significantly modify the quantity of enzymes available from a given amount of transcript. In addition, changes in substrate/product concentrations or metabolic regulations can influence the reaction fluxes irrespective to the enzyme quantities. As a consequence, no simple correlations are necessarily observed between gene expression levels and reaction fluxes (Gygi *et al.*, 1999; ter Kuile & Westerhoff, 2001; Yang *et al.*, 2002; Akesson *et al.*, 2004).

Some approaches have nonetheless been developed to exploit information from gene expression data using models. In the vein of pathway- or module-based methods interpreting changes of gene expressions at the level of pathways or biological processes (Hanisch *et al.*, 2002; Draghici *et al.*, 2003; Yang *et al.*, 2004), methods relying on a graph representation of metabolism (Patil & Nielsen, 2005) or on a decomposition of metabolic models into elementary modes (Schwartz *et al.*, 2007) were introduced to correlate expression levels with possible metabolic states. These approaches are merely descriptive: the model provides a suitable metabolic context to interpret the experimental data. Gene expression data have also been used to refine the characterization of metabolic fluxes in models. For instance, by blocking reactions corresponding to unexpressed genes, metabolic fluxes could be characterized more precisely in a yeast model (Akesson *et al.*, 2004). In the same spirit, a method was recently introduced to evaluate the consistency of gene expression datasets with metabolic objectives, and identify subsets of active reactions that best correlate with expressed genes and metabolic objectives (Becker & Palsson, 2008). Even though these methods only rely on a limited dependency between gene expression level and reaction flux – reactions catalyzed by unexpressed genes should have low fluxes – they succeed in somewhat improving the characterization of metabolic states, or in assessing the consistency of the model with the experimental data.

As an attempt to account for transcriptional regulation, regulatory interactions were introduced in models by translating them into Boolean rules (Covert *et al.*, 2001). In such joint regulatory-metabolic model, Boolean variables qualitatively describe the transcription state of genes, including genes coding for enzymes and transcription factors, while Boolean rules determine their regulatory dependencies. Metabolic reactions are then allowed to have a nonzero flux only if the transcriptional state of their enzymes is *true*. Several methods have been developed to study these joint models. Regulatory FBA (rFBA) simulates time courses of gene expression states: at each time step, the new transcriptional state is computed from the metabolic state predicted at the previous time step, and is used to constrain FBA prediction of the current metabolic state (Covert *et al.*, 2001). A specific representation scheme was later developed to encode the sequence of expression states predicted by rFBA in a unified manner, in order to compare regulatory responses across various environments (Barrett *et al.*, 2005). Another type of method has been recently developed to determine joint steady states of gene expression and metabolic fluxes. Examining these steady states contributed to the identification of redundantly expressed enzymes and the quantification of the effect of transcriptional regulation in determining flux activity in *E. coli* (Shlomi *et al.*, 2007a). Finally, two studies compared experimental expression levels with predicted expression states to assess the correctness of joint regulatory-metabolic models of *E. coli* and yeast (Covert *et al.*, 2004; Herrgård *et al.*, 2006b). A significant proportion of inconsistent expression states could be corrected in these models by searching for missing interactions (Covert *et al.*, 2004; Herrgård *et al.*, 2006b). In the same vein, a method was recently designed to automate the identification of experiments that are likely to bring most information on potentially missing regulatory interactions (Barrett & Palsson, 2006).

### Using genome-scale models for metabolic engineering

The use of microbial organisms for industrial purposes has grown considerably in the past few years, with potential applications ranging from the production of valuable metabolites to the degradation of pollutants and the generation of renewable energy (Janssen *et al.*, 2005; Ro *et al.*, 2006; Peng *et al.*, 2008; Rittmann, 2008). The field of metabolic engineering aims at designing and improving industrial microorganisms through the rational design of genetic manipulations leading to enhanced performance (Bailey, 1991; Stephanopoulos *et al.*, 1998). With the advent of genome-scale experimental technologies, the set of metabolic engineering methods is progressively expanding to include systems-wide analyses, enabling for instance to study the operation of regulatory and metabolic networks at large scale (Park *et al.*, 2008). In this respect, genome-scale metabolic models provide to engineers an effective toolbox to investigate the metabolic behavior of their strain of interest and target improvements (Kim *et al.*, 2008).

As a first class of applications, all analytical methods presented in the previous sections can be directly applied to engineering purposes. Such methods may help for instance to evaluate the maximum theoretical efficiencies of pathways or determine appropriate host strains by predicting their metabolic capabilities from their reconstructed models. More importantly, metabolic models can help in characterizing the actual metabolism operation of engineered strains, especially when experimental data have been acquired on them. Metabolic Flux Analysis provides for instance quantitative values for intracellular fluxes, which may be used to determine the actual pathway utilization and pinpoint bottleneck reactions (Stephanopoulos *et al.*, 1998). Such information is of high significance for the metabolic engineers, as it may help them in designing further metabolic modifications.

Metabolic models also provide the ability to formulate hypotheses and evaluate *in silico* the potential of genetic modifications. A common cause of low production yields lies in the presence of pathways that divert fluxes to the production of undesirable byproducts or compete for the utilization of precursors and cofactors. While such pathways may be identified manually, their direct removal through gene deletion may cause side effects, for example alter the regeneration of cofactors, the redox balance, or the energy balance (Kim *et al.*, 2008). Genome-scale models can predict the effect of gene deletions on metabolic phenotypes. Several methods were designed with the aim of selecting those gene deletions that would provide the greatest benefit for a given metabolite production goal. Alper *et al.* (2005a) developed a procedure that sequentially screen the effect of single and multiple gene deletions in order to select those enabling the best product yields while maintaining sufficient growth rates. They successfully applied their method to enhance the yield of a lycopene producing *E. coli* strain (Alper *et al.*, 2005b). Screening *in silico* the high number of combinations of multiple gene deletions may turn out to be costly and practically impossible. Optimization methods based on genetic (Patil *et al.*, 2005) or linear programming (Burgard *et al.*, 2003) algorithms were introduced to circumvent this issue. The second optimization method, called OptKnock, specifically searches gene deletions coupling the production of a targeted metabolite with growth rate; the rationale being that improving the growth rate by adaptive evolution would jointly improve the metabolite production rate and that this coupling would make the engineered strain more evolutionary stable (Burgard *et al.*, 2003). Gene deletions proposed by this method were tested experimentally to enhance lactic acid production in an *E. coli* strain (Fong *et al.*, 2005). Adaptive evolution experiments performed on the engineered strains actually showed that lactic acid production was coupled to growth and achieved increased secretion rates of the product. In addition to gene deletions, metabolic models can explore the effect of adding new pathways, and help select the most appropriate ones. In this aim, the OptStrain method was designed to systematically suggest additions of reactions to produce novel metabolites (Pharkya *et al.*, 2004). OptStrain relies on a comprehensive database of biochemical reactions and may propose alternative solutions. A last set of methods consists in designing suitable up- or downregulations of metabolic enzymes. Intervening on gene expression levels is indeed a powerful tool to tune metabolism operation, but the specific effects of such interventions are often hardly predictable (Kim *et al.*, 2008). In a study involving a l-threonine producing strain of *E. coli*, Lee *et al.* (2007) made use of its metabolic model to predict gene expression changes enhancing the strain yield. Specifically, they predicted flux values of key reactions leading to optimal l-threonine production and compared them with measured fluxes. They then used the relative difference between them to guide the tuning of the expression of the corresponding genes. A more systematic approach was introduced with the OptReg method, which identifies at genome-scale the relative changes of flux values with respect to the wild-type flux distribution that provide the best production yield (Pharkya & Maranas, 2006). Results of OptReg can be used to identify candidate enzymes for up- or downregulation.

Yet, two main issues limit the predictive capabilities of metabolic models. First, while regulation may play a central role in controlling the efficiency of product synthesis, it is completely overlooked in metabolic models. Studying regulatory interactions – using for instance models of regulatory networks – may actually provide useful insights, for example to remove feedback inhibitions or fine-tune transcriptional regulatory circuits commanding the product biosynthesis (Kim *et al.*, 2008). Not accounting for enzyme quantities but only reaction fluxes imposes a second limitation to genome-scale models. Implementing changes in flux values – suggested for instance by metabolic model optimization methods – by altering the quantity of enzymes is a difficult task, as enzyme kinetics and metabolite concentrations may significantly influence the flux change. In order to determine the effect of enzyme quantity changes on metabolic fluxes, more detailed approaches are required, for example metabolic control analysis (Fell, 1992).

## Resources, databases, and tools

At the time of this review, genome-scale models have been reconstructed for at least 17 bacteria (see Table 4). For all of them, extensive manual curation was required in order to integrate information from the literature on their biochemistry and physiology with functional information from genome annotation. These models are therefore of high quality on average, and mostly complete with respect to the current knowledge of their metabolism. An increasing subset is being assessed and corrected against large-scale experimental data (see Table 4), and an impressive array of analytical studies has been applied to the most popular ones, for example *E. coli* (Feist & Palsson, 2008).

Models used to be made available independently by their authors, under a variety of naming conventions and formats. This is a significant obstacle to their reusability, as significant effort is required to adapt them to modeling software other than the ones they were constructed with. Differences in reaction and metabolite names also hamper direct comparisons between different models. Fortunately, some attempts to address these issues are under way. The general-purpose SBML format (Systems Biology Markup Language) (Hucka *et al.*, 2003) is often used to exchange constraint-based models, thus playing the role of a ‘default’ standard for models. While SBML can be imported by many modeling tools, it is not fully adapted to the specifics of models; this may result in information or functionality loss during exchange. In addition to providing a standard format, SBML supports the association of model components with external references, such as reaction and metabolite identifiers in universal metabolic databases, using MIRIAM annotations (Le Novère *et al.*, 2005). If widely used, this feature should facilitate model reuse and comparison.

In order to facilitate model reuse and comparison, dedicated model repositories have been developed. Perhaps the most widely adopted initiative of this type is the Biomodels.net repository (Le Novère *et al.*, 2006) which stores biochemical models of any type in SBML format. Because of its focus on more detailed dynamic models and the related generic format choice, the repository is not fully compatible with constraint-based models and qualitative predictions, as illustrated by the current low number of such models included. Agreements with several journals make it mandatory for authors to deposit models mentioned in their manuscripts in Biomodels.net, where they are checked for syntactic correctness. On some models, a more elaborate test on the compatibility between model predictions and results presented in the associated paper is also performed.

Currently, the only freely accessible (to academic users) repository dedicated to constraint-based models is the BiGG database (http://bigg.ucsd.edu). Its unified dictionary of metabolite and reaction names enables direct comparisons between its metabolic models.

Relatively few software tools have been specifically developed to handle genome-scale constraint-based models, compared with the number of tools developed for kinetic modeling. As the modeling framework relies primarily on linear algebra and linear programming, general purpose mathematical software platforms, for example matlab (http://www.mathworks.com/) and mathematica (http://www.wolfram.com/), or optimization modeling packages, for example gams (http://www.gams.com/), are well suited. Specialized optimization packages can be added for greater efficiency. In addition, modules dedicated to constraint-based modeling have been developed for matlab: fluxanalyzer (Klamt *et al.*, 2007), the cobra toolbox (Becker *et al.*, 2007), or metatool (von Kamp & Schuster, 2006) for elementary mode analysis are good representatives. Libraries for importing SBML models within these programs are also provided by the SBML developer community (Bornstein *et al.*, 2008). Among the software tools that are stand-alone, one should mention the systems biology research toolbox (Wright & Wagner, 2008), scrumpy (Poolman, 2006), metafluxnet (Lee *et al.*, 2003), or fluxexplorer (Luo *et al.*, 2006), each with their own specific strengths. Interestingly, very few programs focus or even support the model reconstruction process by providing the analytical capabilities for consistency checks: the commercial sympheny platform (http://www.genomatica.com/) associates a metabolic database with several analytical methods, while yanasquare (Schwarz *et al.*, 2007) facilitates the reconstruction of models from KEGG and performs selected structural analyses (e.g. elementary modes). Very recently, web-based tools have been released to enable on-line analyses on specific metabolic models (Beste *et al.*, 2007; Durot *et al.*, 2008). Given the need for faster and better reconstruction, we expect more progress in that direction.

## Concluding remarks and future directions

Constraint-based genome-scale metabolic models can be viewed as ‘systems-level’ analytical layers which enable computation and reasoning on the consequences of the accumulated knowledge on the biochemistry encoded in a given genome, and confrontation of that knowledge with the known physiology of the corresponding species or with additional experimental evidence. These models thus bridge the gap between genotype and phenotype and enable a wide spectrum of analyses and *in silico* experiments, providing a solid foundation for systems analyses and metabolic engineering.

The systematic and automated reconstruction of genome-scale models from genomes and additional high-throughput data may seem like a natural extension of genome annotation (Reed *et al.*, 2006a), but remains beyond the reach of current methods. While genome-scale models can be reconstructed using only sequence and qualitative functional information, gaining the additional predictive and analytical power of models still requires significant effort and expertise. Genome annotations must first be translated into a network, which must then be turned into a model with the help of additional information, and systematically checked with respect to biochemical consistency rules and experimental observations. Only after a model is complete enough to enable meaningful predictions at the phenotypic level can it be used to predict phenotypes or other properties beyond those that can be immediately verified.

Obstacles to automating this process include technical difficulties in translating annotations into proper biochemical activities, and also the fact that methods for model refinement have been designed and applied separately for each type of experimental data. There is increasing pressure for this situation to evolve, however, as the boost in the throughput of experimental techniques and the advent of ‘multi-omics’ datasets (Ishii *et al.*, 2007a) promises a wealth of information that will be exploitable only by computer-assisted interpretation, with the help of models. At the same time, the field of metabolic modeling is now approaching the level of maturity necessary for several data integration methods to be used together as components in integrated model reconstruction and refinement strategies.

Significant benefits could result from the availability of a wider spectrum of bacterial metabolic models. They would provide an integrated view of metabolic pathways across the tree of life, thereby enabling so-called transverse approaches to annotation, and a variety of comparative metabolic analysis. To that end, the notion of pathway – defined unambiguously as the conversion between specified sets of input compounds (reactants) and output compounds (products) – can bring a useful decomposition of metabolism into basic biochemical functional units, in the spirit pioneered by SEED (Overbeek *et al.*, 2005), KEGG Modules (Kanehisa *et al.*, 2007), or MetaCyc (Caspi *et al.*, 2006). The field of bacterial evolution is poised to benefit as well: for instance, the availability of models for several bacteria along the phylogenetic tree would allow more comprehensive studies on the constraints implied by bacteria's metabolic capabilities and their evolution. While this type of study has been pioneered with a few selected models (Pál *et al.*, 2005a, 2006), working with a larger set of models will undoubtedly bring different insights (see (Kreimer *et al.*, 2008) for an example with networks). Modeling can also help in studying bacterial communities, as chemical interactions occurring between bacteria often need to be understood within the context of their metabolisms. Indeed, models have already been reconstructed and analyzed for small communities (Stolyar *et al.*, 2007); progress on that front may prove very useful in studying metabolic interactions in more complex communities, assisting in the functional interpretation of metagenome sequences. Last but not least, metabolic engineering applications would clearly benefit from the availability of a large set of bacterial models, as these would constitute a repository of characterized metabolic pathways, facilitating the combinatorial design of new catalytic systems, providing solid bases to test hypothetical genetic constructions, and helping with the selection of relevant strains for specific engineering objectives.
